# Comparison of Clinical and Pathological Factors Affecting Early and Late Recurrences in Patients with Operable Breast Cancer †

**DOI:** 10.3390/jcm11092332

**Published:** 2022-04-22

**Authors:** Emre Yekedüz, Ömer Dizdar, Neyran Kertmen, Sercan Aksoy

**Affiliations:** 1 Department of Internal Medicine, Hacettepe University School of Medicine, Ankara 06230, Turkey; 2 Department of Medical Oncology, Hacettepe University School of Medicine, Ankara 06230, Turkey; dromerdizdar@gmail.com (Ö.D.); neyran_kertmen@yahoo.com (N.K.); saksoy07@yahoo.com (S.A.)

**Keywords:** breast cancer, early recurrence, late recurrence, disease-free survival

## Abstract

In this study, we aimed to assess clinicopathological factors affecting early and late recurrences in patients with operable breast cancer. Patients with early (≤5 years) and late (>5 years) recurrences were assessed. Prognostic factors for disease-free survival (DFS) were also evaluated in patients with recurrence. A total of 854 patients were included. There were 432 and 205 patients in the early and late recurrence groups, respectively. In multivariate analyses, HER2+ disease, lymph node metastasis, lymphovascular invasion (LVI), and high tumor grade were associated with increased risk of early recurrence, while HER2+ disease and LVI were associated with decreased risk of late recurrence. In multivariate analyses, presence of HER2+ disease and triple-negative breast cancer (TNBC) were poor prognostic factors for DFS in patients with early recurrence. Presence of LVI and perineural invasion (PNI) were poor prognostic factors for DFS in patients with late recurrence. Molecular subtypes and LVI were effective on the early and late recurrences. However, lymph node positivity and grade were only associated with the early recurrence. After 5 years, LVI and PNI were the prognostic factors for DFS.

## 1. Introduction

Breast cancer is the most common cancer in women [1]. According to the Surveillance, Epidemiology, and End Results (SEER) database, the 5-year survival rate is 90.3% [2]. The most significant problem in patients with breast cancer treated with surgery is the recurrence of the disease after treatment. Recurrence is usually seen in the first 5 years, particularly within the first and second years [3]. The annual recurrence risk for the first 5 years was reported as 10.4% [3]. It has been shown that the recurrence and mortality rates decreased until 10 years and continued at a fixed rate thereafter [3].

When the patient groups are evaluated separately, there are conflicting results in the literature regarding recurrence in the first 5 years and after 5 years. In one study, estrogen receptor (ER)-negative patients were more likely to have a recurrence in the first 5 years and ER-positive patients were more likely to have a recurrence after 5 years [3]. In another study, progesterone receptor (PR)-positivity and lymph node involvement were associated with late recurrence [4]. ER-positivity was associated with late recurrence after 10 years [5]. Large and lymph node-positive tumors had a higher recurrence rate in the first 5 years while ER and PR-positive/human epidermal growth factor receptor 2 (HER2)-negative tumors were the risk factors for late recurrence after 5 years [6]. Considering the annual incidence of recurrence, the increase in follow-up interval after 5 years may lead to delayed diagnosis of recurrences in patients with a high risk of late recurrence. Closer follow up may be needed in a subgroup of patients with a high risk of late recurrence after 5 years. 

In this study, we aimed to assess clinicopathological factors associated with early and late recurrences in a large group of patients with early breast cancer.

## 2. Materials and Methods

This retrospective study was conducted in a tertiary cancer center in Turkey. Adult patients diagnosed with early breast cancer from inception to January 2018 were included in the study and categorized according to the presence and time of recurrence; early recurrence (≤5 years), late recurrence (>5 years). Patients treated with neoadjuvant therapy and those with bilateral breast cancer were excluded. Radiological and pathological recurrences were accepted as cancer recurrence.

All clinical (age, menopausal status, obesity, date of diagnosis, surgery and recurrence, recurrence sites) and pathological (tumor size, nodal involvement, lymphovascular invasion (LVI), perineural invasion (PNI), tumor grade, type of surgery, adjuvant treatment) data were extracted from the electronic hospital records of the patients.

The median with interquartile range (IQR) and percentages were used to define the continuous and categorical variables, respectively. In univariate analysis, the chi-square test was used to determine the risk factors for early and late recurrence. Variables found to be statistically significant in univariate analysis were analyzed by using binary logistic regression models and adjusted odds ratio (aOR) was calculated. Disease-free survival (DFS) was calculated from surgery to disease recurrence. Prognostic factors for DFS in each recurrence interval (early and late) were evaluated by using Kaplan–Meier estimates and survival curves were compared by using the log-rank test. Variables found to be statistically significant in univariate analyses were analyzed by using Cox’s regression models. A *p*-value less than 0.05 was considered statistically significant for all statistical analyses. All analyses were done by using the SPSS 28.0 for Mac (IBM Corp., Armonk, NY, USA) and RStudio (RStudio, Boston, MA, USA).

## 3. Results

### 3.1. Baseline Characteristics

A total of 854 patients with early breast cancer were included in this study. The median age was 45 (IQR: 39–53) years. The majority of patients had ductal histology (83.4%) and hormone-receptor-positive disease (70.5%). The node-negative disease was observed in 37.5% of all patients. A total of 100 (11.7%) did not receive adjuvant chemotherapy. Median follow-up was 179 months (95% Confidence Interval (CI): 171–186). There were 637 and 217 patients with and without recurrence, respectively. A total of 68% of the patients (432 out of 637) in the recurrence group had early recurrence (≤5 years), while the remaining (n = 205) had late recurrence (>5 years). About 20% of all patients had a locoregional recurrence. Baseline characteristics of patients are shown in Table 1.

### 3.2. Risk Factors for Early and Late Recurrences 

In univariate analyses, younger patients (≤35 years old) (OR: 1.61, 95% CI: 1.11–2.33, *p* = 0.012), patients with ductal histology (OR: 1.48, 95% CI: 1.01–2.18, *p* = 0.049), HER2+ disease (OR: 2.69, 95% CI: 1.82–3.99, *p* < 0.001), triple-negative breast cancer (TNBC) (OR: 1.53, 95% CI: 1.01–2.32, *p* = 0.045), larger tumor (T3–4) (OR: 1.73, 95% CI: 1.20–2.49, *p* = 0.003), lymph node metastasis (OR: 2.50, 95% CI: 1.87–3.34, *p* < 0.001), LVI (OR: 3.77, 95% CI: 2.69–5.29, *p* < 0.001), PNI (OR: 2.50, 95% CI: 1.43–4.39, *p* = 0.001), high-grade tumor (grade 2 or 3) (OR: 3.09, 95% CI: 1.87–5.12, *p* < 0.001), and breast-conserving surgery (OR: 1.44, 95% CI: 1.03–2.01, *p* = 0.030) had a higher risk of early recurrence. On the other hand, late recurrence risk was lower in patients with HER2+ disease (OR: 0.47, 95% CI: 0.28–0.77, *p* = 0.003) and with LVI (OR: 0.43, 95% CI: 0.28–0.66, *p* < 0.001). Obesity and menopausal status were not associated with recurrence period (Table 2).

In multivariate analyses, HER2+ disease (aOR: 1.74, 95% CI: 1.10–2.75, *p* = 0.017), lymph node metastasis (aOR: 1.66, 95% CI: 1.12–2.47, *p* = 0.011), LVI (aOR: 2.40, 95% CI: 1.59–3.63, *p* < 0.001), and high tumor grade (aOR: 2.46, 95% CI: 1.33–4.52, *p* = 0.004) were associated with increased risk of early recurrence, while HER2+ disease (aOR: 0.49, 95% CI: 0.29–0.84, *p* = 0.010) and LVI (aOR: 0.49, 95% CI: 0.31–0.76, *p* = 0.001) were associated with decreased risk of late recurrence after adjusting for confounding variables (Table 3).

### 3.3. Prognostic Factors for Disease-Free Survival According to Recurrence Interval

In the early recurrence group (≤5 years), the median DFS was longer in patients with hormone-receptor-positive/HER2-negative breast cancer than in patients with HER2+ disease and TNBC (31.8, 22.1, and 19.4 months in the hormone-receptor-positive/HER2-negative, HER2+, and TNBC groups, respectively; *p* < 0.001). In addition, presence of LVI (22.9 vs. 29.5 months, *p* < 0.001) was associated with shorter DFS in patients with early recurrence. In the late recurrence group (>5 years), the median DFS was shorter in patients with lymph node metastasis (102.8 vs. 103.9 months, *p* = 0.046), with LVI (83.7 vs. 111.7 months, *p* < 0.001), and with PNI (77.9 vs. 103.9 months, *p* = 0.046). (Table 4) 

**Figure 1:**
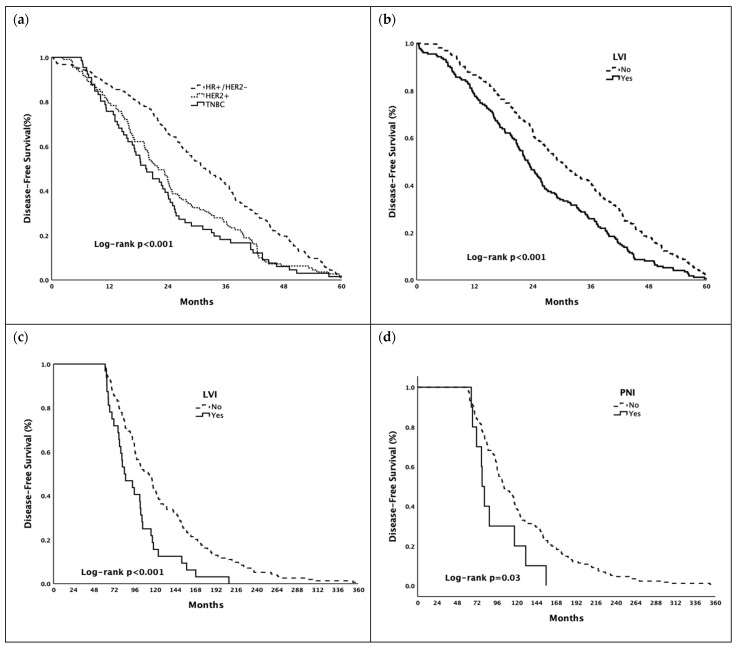
Kaplan–Meier estimates of disease-free survival ((**a**,**b**) in early recurrence group, (**c**,**d**) in late recurrence group). Abbreviations: HR—Hormone Receptor; HER2—Human Epidermal Growth Factor Receptor 2; LVI—Lymphovascular Invasion; PNI—Perineural Invasion; TNBC—Triple-Negative Breast Cancer.

Survival curves for DFS in early and late recurrence groups are shown in Figure 1.

In multivariate analyses, the presence of HER2+ disease (HR: 1.37, 95% CI: 1.09–1.73, *p* = 0.007) and TNBC (HR: 1.64, 95% CI: 1.25–2.17, *p* < 0.001) were poor prognostic factors for DFS in patients with early recurrence (≤5 years) after adjusting for confounding variables. Presence of LVI (HR: 1.79, 95% CI: 1.19–2.69, *p* = 0.005) and PNI (HR: 1.99, 95% CI: 1.04–3.81, *p* = 0.036) were poor prognostic factors for DFS in patients with late recurrence (>5 years) after adjusting for confounding variables. (Table 5).

## 4. Discussion

Clinical and pathological features of patients with breast cancer are easily accessible variables to define the risk of recurrence in operable patients after surgery. To the best of our knowledge, this study includes one of the largest patient numbers in the literature. In this study, we showed that HER2+ disease, lymph node involvement, presence of LVI, and higher tumor grade were the risk factors for early recurrence. On the other hand, the risk of late recurrence was lower in patients with HER2+ disease and those with LVI. When we looked at the prognostic factors, patients with HER2+ disease or TNBC and those with LVI had a poorer prognosis in the early recurrence group. Additionally, the median DFS was shorter in patients with LVI and those with PNI in the late recurrence group. 

ER and/or PR positivity is one of the most important prognostic markers. They have not only prognostic but also predictive values [7]. It has been known for many years that ER positivity has a positive effect on the prognosis, especially in the early periods [8]. In ER-negative tumors, although the risk of recurrence is high in the early period, it was observed that this effect was absent in the late periods [9]. However, there are conflicting data regarding the effect of ER-positive or negative status on late prognosis. There are also publications showing that prognosis is negatively affected in patients with ER positivity after 5 years [3]. HER2 status is another prognostic and predictive marker. In a study comparing early and late recurrences, ER/PR-positive and HER2-negative patients were observed to have a higher recurrence risk after 5 years [6]. For HER2-positive tumors, the reverse situation is observed. Early recurrence has been shown to be more frequent in this patient group [10]. In our study, the risk of early recurrence was higher in patients with HER2+ disease. 

The pathological grade is a prognostic factor in breast cancer [11,12]. When recurrence periods are studied separately, studies concluded that grade is effective on recurrences after 5 years [6]. Conversely, there are also studies showing that grade is effective on recurrences in the first 5 years [13,14]. In our study, it was observed that the grade was a risk factor for recurrences in the first 5 years. 

LVI increases the risk of recurrence in patients with early breast cancer [15,16]. Similarly, in our study, high early and late recurrence rates were observed in patients with LVI. On the other hand, there were conflicting data in the literature on the effect of LVI on DFS and recurrence periods. A study comparing early and late recurrences stated that the patients with LVI experienced early recurrence [6]. In another study, however, there was no difference in recurrence rates before and after 10 years in patients with LVI [17]. A study assessing late recurrences in patients with early-stage breast cancer stated that DFS was shorter in the patient group with LVI in recurrences after 10 years [5]. In our study, the median DFS was observed to be shorter in patients with LVI in recurrences in the first 5 years compared to the group without LVI. Furthermore, this effect was also observed in recurrences after 5 years. Since PNI is not common in cases of breast cancer, data on recurrence and survival rates are limited in the literature. However, there are studies showing that DFS rates decrease in the presence of PNI [18]. On the other hand, there are also publications showing that PNI has no significant effect on DFS [19]. In our study, presence of PNI was associated with shorter DFS in the late recurrence group. 

Survival rates decrease with increasing tumor size [20]. Studies comparing the effect of the tumor size according to recurrence period have shown that large tumor size causes early recurrences [6,20,21]. There are publications showing that tumor size affects recurrence in 5–10 years [22]. Several studies stated that tumor size and T stage do not affect DFS in late recurrences, especially after 10 years [4,5]. In our study, we demonstrated no effect of the T stage on the recurrence intervals. One of the most important prognostic markers in breast cancer is the N stage, which is determined according to local and axillary lymph node involvement. In terms of the N stage, the prognosis deteriorates as the stage progresses [23]. In terms of the recurrence period, early recurrence is more frequent in patients with lymph node involvement [6,24]. However, there are studies showing that lymph node positivity is a risk factor for late recurrences after 10 years [4,24]. In our study, it was observed that node-positive tumors were higher risk of early recurrence. 

Because breast cancer is a hormone-dependent tumor, menopausal states of patients play an important role in the progression of the disease. In a study analyzing the relationship between menopausal states of patients and recurrence period, lymph node-positive premenopausal patients showed to have an earlier recurrence [25]. In another study comparing early and late recurrences, there was no statistically significant difference between premenopausal and postmenopausal patient rates [6]. In our study, there was no effect of menopausal status on the risk of early and late recurrence and DFS.

Obesity is considered a risk factor, especially since postmenopausal women are likely to have increased exposure to estrogen [26]. It has been proven by many studies that patient groups with obesity have both a high recurrence rate and a shorter DFS [27,28,29]. However, in our study, there was no effect of obesity on the risk of early and late recurrence and DFS.

Our study has several limitations due to its retrospective nature. First, because treatment options for the patients were planned at the start of the treatment according to the patient and tumor characteristics and retrospective randomization was not an option for patients, it was considered to have no statistical value to analyze and compare DFS according to recurrence periods of treatment regimens. Second, in our study, molecular subtypes were studied in three groups: hormone receptor-positive/HER2-negative, HER2-positive, and TNBC. Because the follow-up time of patients was long, the Ki-67 proliferative index could not be obtained for patients whose pathological data were old. Due to the lack of data about Ki-67, patients were grouped into three subgroups. Third, we did not evaluate local and distant recurrence risks separately. In a study of assessing the late distant recurrence risk in patients with hormone receptor-positive breast cancer showed that age, nodal involvement, tumor size and grade could be used to calculate a risk score, named as “Clinical Treatment Score Post 5 Years” [30].

## 5. Conclusions

Our study showed that the most frequent recurrences occurred in the first 5 years in patients with operable breast cancer. Molecular subtypes and LVI were effective on the early and late recurrences. However, lymph node positivity and grade were only associated with the early recurrence. After 5 years, LVI and PNI were the prognostic factors for DFS. Because of this reason, patients with PNI and/or LVI should be followed carefully after 5 years from the surgery. Clinical and pathological factors in our study are easily accessible to determine the prognosis and recurrence risk in patients with early breast cancer. However, prospective studies including the molecular profile of those patients might present more precise information about the risk of recurrence.

## Figures and Tables

**Table 1:** Baseline characteristics of all patients.

	All Patients		Early Recurrence (≤5 years)		Late Recurrence (>5 years)	
	n = 854	(%)	n = 432	(%)	n = 205	(%)
**Age at diagnosis-years, median (IQR)**	45 (39–53)		45 (37–54)		45 (39–52)	
**Histological Subtypes**						
Invasive Ductal Carcinoma	712	83.4	378	87.5	165	80.5
Invasive Lobular Carcinoma	40	4.7	12	2.8	14	6.8
Other	80	9.4	40	9.3	15	7.3
Missing	22	2.6	2	0.5	11	5.4
**Molecular Subtypes**						
HR+/HER2−	509	59.6	249	57.6	128	62.4
HER2+	154	18.0	111	25.7	21	10.3
Triple Negative	111	13.0	66	15.3	22	10.8
Missing	80	9.4	6	1.4	34	16.5
**T Stage**						
T1	173	20.3	64	14.8	49	23.9
T2	464	54.3	259	60.0	101	49.3
T3	130	15.2	79	18.3	24	11.7
T4	23	2.7	19	4.4	3	1.5
Missing	64	7.5	11	2.5	28	22.6
**N Stage**						
N0	320	37.5	121	28.0	75	36.6
N1	213	24.9	110	25.5	58	28.3
N2	120	14.1	65	15.0	29	14.1
N3	164	19.2	125	28.9	28	13.7
Missing	37	4.3	11	2.5	15	7.3
**Lymphovascular Invasion**						
No	577	67.6	253	58.6	154	75.1
Yes	233	27.3	174	40.3	32	15.6
Missing	44	5.2	5	1.2	19	9.3
**Perineural Invasion**						
No	745	87.2	380	88.0	176	85.9
Yes	65	7.6	47	10.9	10	4.9
Missing	44	5.2	5	1.2	19	9.3
**Grade**						
1	75	8.8	26	6.0	19	9.3
2	294	34.4	161	37.3	61	29.8
3	297	34.8	208	48.1	47	22.9
Missing	188	22.0	37	8.6	78	38.0
**Menopausal Status**						
Pre-	523	61.2	269	62.3	117	57.0
Post-	328	38.4	162	37.5	86	42.0
Missing	3	0.4	1	0.2	2	1.0
**Obesity**						
No	575	67.3	276	63.9	132	64.4
Yes	190	22.2	103	23.8	52	25.4
Missing	89	10.4	53	12.3	21	10.2
**Type of Surgery**						
Modified Radical Mastectomy	674	78.9	328	75.9	167	81.5
Breast Conserving Surgery	180	21.1	104	24.1	38	18.5
**Adjuvant Chemotherapy**						
No	100	11.7	37	8.6	30	14.6
Yes	731	85.6	387	89.6	164	80.0
Missing	23	2.7	8	1.9	11	5.4
**Adjuvant Hormonal Therapy**						
No	264	30.9	134	31.0	60	29.3
Yes	586	68.6	295	68.3	145	70.7
Missing	4	0.5	3	0.7	0	0
**Adjuvant HER-2 Targeted Therapy**						
No	745	87.2	350	81.0	189	92.2
Yes	86	10.1	74	17.1	5	2.4
Missing	23	2.7	8	1.9	11	5.4
**Adjuvant Radiotherapy**						
No	264	30.9	96	22.2	72	35.1
Yes	588	68.9	336	77.8	132	64.4
Missing	2	0.2	0	0	1	0.5
**Recurrence Locations**						
Locoregional	168	19.7	88	20.4	80	39.0
Distant	470	55.0	344	79.6	125	61.0
Missing	216	25.3	0	0	0	0

Abbreviations: HR—Hormone-Receptor; HER-2—Human Epidermal Growth Factor Receptor-2; IQR—Interquartile Range.

**Table 2:** Univariate analysis of risk factors for early and late recurrence.

	Early Recurrence (≤5 years)		*p* Value	Late Recurrence (>5 years)		*p* Value
	OR	95% CI		OR	95% CI	
**Age**			**0.012**			0.245
≤35	1.61	1.11–2.33		1		
>35	1			1.30	0.83–2.04	
**Histological Subtypes**			**0.049**			0.812
Ductal	1.48	1.01–2.18		0.94	0.60–1.48	
Non-ductal	1			1		
**Molecular Subtypes**						
HR+/HER2−	1			1		
HER2+	2.69	1.82–3.99	**<0.001**	0.47	0.28–0.77	**0.003**
Triple Negative	1.53	1.01–2.32	**0.045**	0.73	0.44–1.22	0.236
**T Stage**			**0.003**			0.117
T12	1			1		
T34	1.73	1.20–2.49		0.69	0.44–1.09	
**Lymph Node Metastasis**			**<0.001**			0.921
No	1			1		
Yes	2.50	1.87–3.34		0.98	0.70–1.37	
**Lymphovascular Invasion**			**<0.001**			**<0.001**
No	1			1		
Yes	3.77	2.69–5.29		0.43	0.28–0.66	
**Perineural Invasion**			**0.001**			0.134
No	1			1		
Yes	2.50	1.43–4.39		0.58	0.29–1.17	
**Grade**			**<0.001**			0.153
1	1			1		
2/3	3.09	1.87–5.12		0.66	0.37–1.16	
**Menopausal Status**			0.562			0.200
Pre-	1			1		
Post-	0.92	0.69–1.21		1.23	0.89–1.69	
**Obesity**			0.138			0.218
No	1			1		
Yes	1.28	0.92–1.78		1.26	0.87–1.83	
**Surgery Type**			**0.030**			0.307
Mastectomy	1			1		
Breast Conserving	1.44	1.03–2.01		1.23	0.82–1.83	

Abbreviations: HR—Hormone-Receptor; HER-2—Human Epidermal Growth Factor Receptor-2.

**Table 3:** Multivariate analysis of risk factors for early and late recurrence.

	Early Recurrence (≤5 years)		*p* Value	Late Recurrence (>5 years)		*p* Value
	aOR *	95% CI		aOR *	95% CI	
**Age**			0.137			
≤35	1.46	0.88–2.40				
>35	1					
**Histological Subtypes**			0.377			
Ductal	1.27	0.74–2.16				
Non-ductal	1					
**Molecular Subtypes**						
HR+/HER2−	1			1		
HER2+	1.73	1.10–2.72	**0.018**	0.49	0.29–0.84	**0.010**
Triple Negative	1.42	0.86–2.34	0.170	0.76	0.45–1.28	0.310
**T Stage**			0.363			
T12	1					
T34	1.23	0.78–1.96				
**Lymph Node Metastasis**			**0.009**			
No	1					
Yes	1.67	1.13–2.48				
**Lymphovascular Invasion**			**<0.001**			**0.001**
No	1			1		
Yes	2.42	1.61–3.63		0.49	0.31–0.76	
**Perineural Invasion**			0.279			
No	1					
Yes	1.43	0.74–2.77				
**Grade**			**0.014**			
1	1					
2/3	2.09	1.16–3.77				
**Surgery**			0.166			
Mastectomy	1					
Breast Conserving	1.36	0.87–2.12				

Abbreviations: aOR—Adjusted Odds Ratio; HR—Hormone-Receptor; HER-2—Human Epidermal Growth Factor Receptor-2; * aOR was calculated by using binary logistic regression analysis.

**Table 4:** Univariate analysis of disease-free survival.

	Early Recurrence (≤5 years)		Late Recurrence (>5 years)	
	Months	*p*	Months	*p*
**Age**		0.993		0.053
≤35	32.2		118.2	
>35	30.5		103.9	
**Histological Subtypes**		0.963		0.191
Ductal	26.1		104.6	
Non-ductal	29.1		97.2	
**Molecular Subtypes**		**<0.001**		0.185
HR+/HER2−	31.8		98.1	
HER2+	22.1		91.8	
Triple Negative	19.4		106.5	
**T Stage**		0.472		0.523
T12	26.3		98.2	
T34	26.4		117.5	
**Lymph Node Metastasis**		0.753		**0.046**
No	28.2		103.9	
Yes	25.9		102.8	
**Lymphovascular Invasion**		**<0.001**		**<0.001**
No	29.5		111.7	
Yes	22.9		83.7	
**Perineural Invasion**		0.996		**0.030**
No	26.1		103.9	
Yes	29.1		77.9	
**Grade**		0.314		0.806
1	33.4		95.6	
2/3	26.1		93.3	
**Menopausal Status**		0.668		0.600
Pre-	26.4		111.1	
Post-	27.0		102.4	
**Obesity**		0.380		0.075
No	26.1		113.1	
Yes	27.0		102.8	
**Surgery Type**		0.186		0.494
Mastectomy	26.6		113.1	
Breast Conserving	26.1		97.2	

Abbreviations: HR—Hormone-Receptor; HER-2—Human Epidermal Growth Factor Receptor-2.

**Table 5:** Multivariate analysis of disease-free survival.

	Early Recurrence (≤5 years)			Late Recurrence (>5 years)		
	Hazard Ratio *	95% CI	*p*	Hazard Ratio *	95% CI	*p*
**Molecular Subtypes**						
HR+/HER2−	1					
HER2+	1.37	1.09–1.73	**0.007**			
Triple Negative	1.64	1.25–2.17	**<0.001**			
**Lymph Node Metastasis**						0.200
No				1		
Yes				1.23	0.89–1.69	
**Lymphovascular Invasion**			**0.001**			**0.005**
No	1			1		
Yes	1.38	1.13–1.69		1.79	1.19–2.69	
**Perineural Invasion**						**0.036**
No				1		
Yes				1.99	1.04–3.81	

Abbreviations: HR—Hormone-Receptor; HER-2—Human Epidermal Growth Factor Receptor-2; * Hazard ratio was calculated by using Cox’s regression analysis.

## Data Availability

The data that support the findings of this study are not publicly available but are available from the corresponding author [E.Y.] upon reasonable request.
